# Structural Studies of the HIV-1 Integrase Protein: Compound Screening and Characterization of a DNA-Binding Inhibitor

**DOI:** 10.1371/journal.pone.0128310

**Published:** 2015-06-05

**Authors:** Peter K. Quashie, Ying-Shan Han, Said Hassounah, Thibault Mesplède, Mark A. Wainberg

**Affiliations:** 1 McGill University AIDS Centre, Lady Davis for Medical Research, Jewish General Hospital, Montreal, Quebec, Canada; 2 Division of Experimental Medicine, Faculty of Medicine, McGill University, Montreal, Quebec, Canada; 3 Department of Microbiology and Immunology, Faculty of Medicine, McGill University, Montreal, Quebec, Canada; QIMR Berghofer Medical Research Institute, AUSTRALIA

## Abstract

Understanding the HIV integrase protein and mechanisms of resistance to HIV integrase inhibitors is complicated by the lack of a full length HIV integrase crystal structure. Moreover, a lentiviral integrase structure with co-crystallised DNA has not been described. For these reasons, we have developed a structural method that utilizes free software to create quaternary HIV integrase homology models, based partially on available full-length prototype foamy virus integrase structures as well as several structures of truncated HIV integrase. We have tested the utility of these models in screening of small anti-integrase compounds using randomly selected molecules from the ZINC database as well as a well characterized IN:DNA binding inhibitor, FZ41, and a putative IN:DNA binding inhibitor, HDS1. Docking studies showed that the ZINC compounds that had the best binding energies bound at the IN:IN dimer interface and that the FZ41 and HDS1 compounds docked at approximately the same location in integrase, i.e. behind the DNA binding domain, although there is some overlap with the IN:IN dimer interface to which the ZINC compounds bind. Thus, we have revealed two possible locations in integrase that could potentially be targeted by allosteric integrase inhibitors, that are distinct from the binding sites of other allosteric molecules such as LEDGF inhibitors. Virological and biochemical studies confirmed that HDS1 and FZ41 share a similar activity profile and that both can inhibit each of integrase and reverse transcriptase activities. The inhibitory mechanism of HDS1 for HIV integrase seems to be at the DNA binding step and not at either of the strand transfer or 3' processing steps of the integrase reaction. Furthermore, HDS1 does not directly interact with DNA. The modeling and docking methodology described here will be useful for future screening of integrase inhibitors as well as for the generation of models for the study of integrase drug resistance.

## Introduction

**Fig 1:**
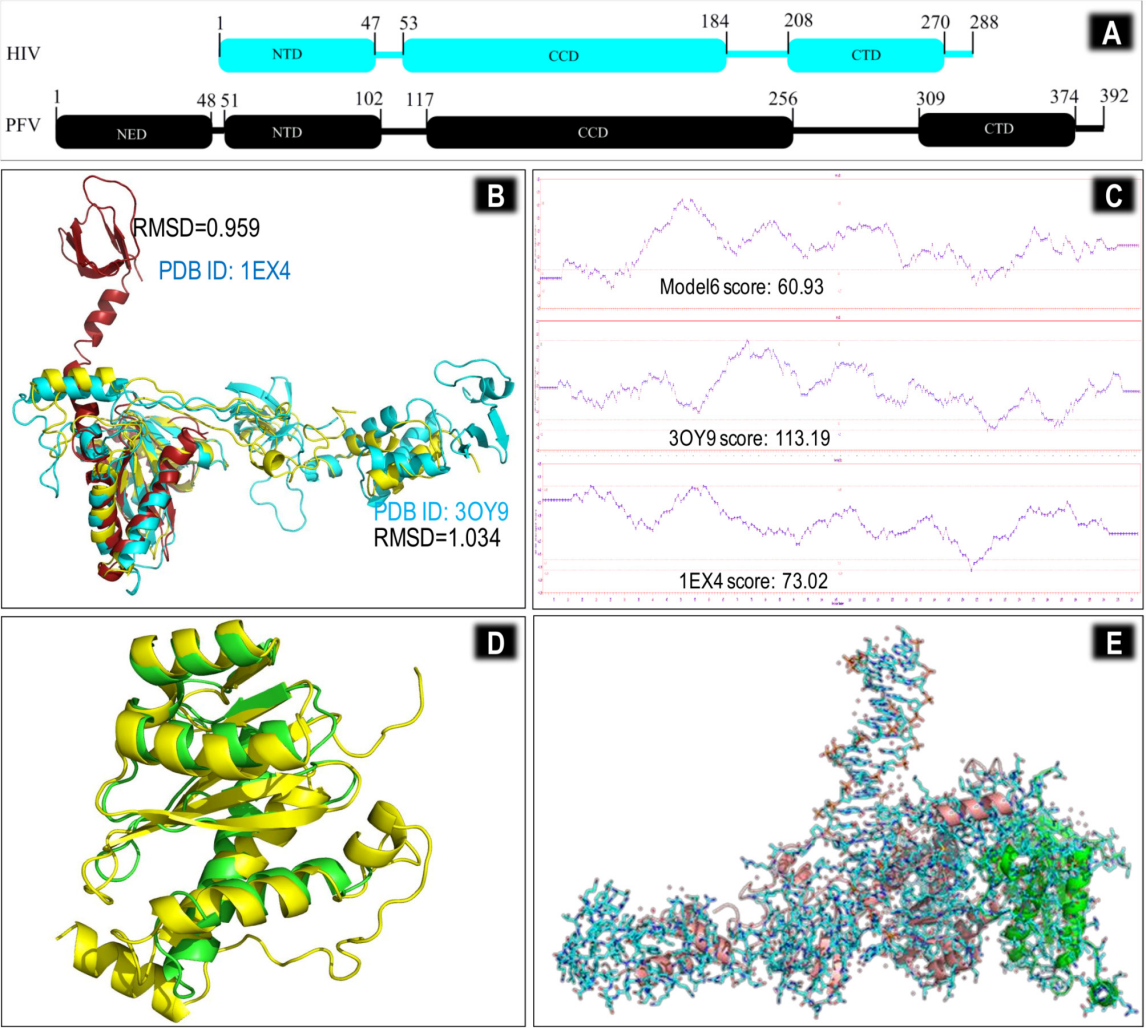
Analysis of model 6 and creation of the dimeric model 7 HIV-1 CRF02_AG IN structure. (A) Domain organization of HIV-1 and PFV IN domains showing regions of structural and sequence overlap as well as structural gaps. (B) Structural alignment of modeled chain A (yellow) aligned with templates 3OY9 (cyan) and 1EX4 (dark green); (C) Comparative Verify3D analysis of the two templates with model 6; (D) Modeled Chain B (yellow) is aligned to PFV chain B (light green) and non-overlapping segments have been removed; (E) Dimeric CRF02_AG IN structure (pink and green cartoon) overlays well with the dimeric PFV structure (shown as sticks) with a global RMSD <1.5Å. With the exception of carbon atoms, all coloration of the PFV stick structure is based on the Corey-Pauling-Koltun (CPK) coloration scheme [18]; white for hydrogen, blue for nitrogen, and red for oxygen.

HIV-1 integrase (IN) is a multi-domain protein that is activated after cleavage from the HIV Gag-Pol poly-protein by HIV protease during viral maturation. HIV IN has three well characterised domains (Fig 1A); an N-terminal dimerization domain (NTD) that has a conserved HCCH Zn^2+^-binding motif, a central RNAse H-like catalytic core domain (CCD), and a C-terminal domain (CTD) that plays a role in IN DNA binding [1–3]. Each of these domains has been purified, crystallised and characterized, either individually, in complex with other proteins, or as double-domain partial structures [1–4]. However, crystallization of the full-length HIV-1 IN structure has been elusive and none of the HIV-1 double-domain partial structures has been crystallized together with DNA. Due to high structural flexibility of IN, the available partial crystal structures are unreliable predictors of HIV-IN inter-monomer interactions and IN-DNA interactions [5]. The coordination of divalent Mg^2+^/Mn^2+^ ions by the D_64_D_116_E_152_ residues is critical for IN activity [6] and this has led to the development of the cation-chelating diketoacid derivative compounds [7, 8] that are currently used as IN strand transfer inhibitors (INSTIs), such as raltegravir (RAL) [9] and elvitegravir (EVG) [10]. Additional structural knowledge was gained through the elucidation of drug resistance mutations for RAL and EVG in tissue culture [11, 12] and clinical trials [13]. However, it was really the successful crystallization of the prototype foamy virus (PFV) IN protein [14–17] that provided an understanding of the correct binding mode of INSTIs and resistance to them(4,18–25).

Unlike crystal structures, homology models are not usually deposited into online servers for universal use so different groups have had to generate their own model(s) [19–21] and validate them, often using molecular dynamics approaches which are beyond the computing abilities of most research groups. Therefore, we have developed a protocol for generation of situation-specific HIV IN models for compound screening or investigation of drug resistance substitutions using free online modeling servers and free software. We have previously used this methodology to model IN proteins of HIV-1 subtype B [22–25], subtype C, and circulation recombinant form number 2 AG (CRF02_AG).

Here, HIV-1 circulating recombinant form number 2 A/G (CRF02_AG) IN was modeled and used to screen for possible inhibitors of IN dimerization or DNA binding.

## Materials and Methods

### Generation of monomeric IN model

Due to the incomplete nature of HIV-1 structures in the PDB, the generation of the initial HIV monomer had to be done by multiple template modeling (MTM) [26]. The sequence of CRF02-A/G IN was submitted to three servers for sequence alignment and homology modeling, HHpred [27], PHYRE2 [28]and I-TASSER [29]. HHpred (hidden homology prediction) is a free online server from the Max-Planck Institute for Biotechnology (http://toolkit.tuebingen.mpg.de/HHpred). It uses comparative hidden Markov statistical models (HMM) [30] to assess amino acid sequence homology and predict protein structure [31] by scanning the query sequence against protein sequence alignment databases such as Pfam (Protein family) [32] and SMART (Simple Modular Architecture Research Tool) [33, 34] (S1 Fig). PHYRE2 (Protein Homology/analogY Recognition Engine v2.0) is an online server developed and maintained by the structural bioinformatics group at Imperial College, London (http://www.sbg.bio.ic.ac.uk/phyre2/html/page.cgi?id=index) [28]. PHYRE2 identifies the structural folding patterns of a query protein by scanning it against a library of known protein structures from the Structural Classification of Proteins Database (SCOP) [35] and the Protein Data Bank (PDB) [36]. I-TASSER (Iterative Threading ASSEmbly Refinement) is an advanced protein homology algorithm which is available as an online server through the ZhangLab server at the University of Michigan (http://zhanglab.ccmb.med.umich.edu/I-TASSER/) [29, 37, 38]. I-TASSER uses multiple individual programs and steps as well as molecular dynamics to create protein structural models of a submitted protein sequence [37]. I-TASSER has been consistently ranked as the best server for online protein structure prediction in the last five competitions of the community-wide experiment Critical Assessment of techniques for protein Structural Prediction (CASP7-CASP11) [38]. CASP rankings are considered the most important metric of method/program confidence in structural biology.

Homology models were created using HHpred by three methods. For model 1, fully automated use of HHpred was used to select templates and construct a structure using MODELLER [39]. In model 2, the HIV-1 template 1EX4 [40] and the PFV template 3OY9 [14] were chosen as templates for modeling by MODELLER. For model 3, the alignment between CRF02_A/G and 3OY9 was used to drive the modeling. PHYRE2 was used in intensive mode and two additional models were thereby derived, i.e. a consensus model (model 5) as well as model 5 that was built by direct alignment with the PFV crystal structure 3OY9 (14). Finally for I-TASSER, the 3OY9 hybrid was used as a lead template to create a final test model (Model 6).

### Verification of model quality and creation of model 7

Main-chain atoms of the models created by the three methods were structurally evaluated using Verify3D [41] and ANOLEA [42]. Briefly, Verify3D compares a three dimensional structure against its own sequence and scores the likelihood of each residue being in its structural class (helix, fold, turn, beta strand, loop, etc), based on the intrinsic properties of that particular amino acid. Good structures have very high scores and improbable structures have low scores [43]. ANOLEA (Atomic Non-Local Environment Assessment), measures the energy for each heavy atom in the structure and performs a pair-wise comparison to the energy of the same heavy atom when present in a non-local environment [44]. Ramachandran analysis was performed using the RAMPAGE server (http://mordred.bioc.cam.ac.uk/~rapper/rampage.php). Ramachandran plots analyse the stereochemistry of amino acid side-chains around a peptide bond and each amino acid side-chain is scored based on angular orientation around the PSI (Ψ -torsion angle of β-carbon and main-chain nitrogen around the α-carbon) and PHI (Φ- torsion angle between β-carbon and main-chain carbonyl carbon) [45]. Since there are a limited number of favourable orientations that can occur for each amino acid, structures can be assessed quickly [45].

When necessary, sequence alignments were edited in an effort to increase the accuracy of modeling. The individual monomers were also aligned with the template structures to verify their structural deviation from the original templates as well as their similarity to the PFV template. One final model was used as a lead template for subsequent models. The ProtMod server (http://ffas.burnham.org/protmod-cgi/protModHome.pl) was used to minimize stochastic error between individual models and remove any sampling errors that may have been introduced by the multi-template modeling method [26] of I-TASSER. Where necessary, side-chain orientations were optimized [46]. Briefly, single template: query (WT: WT/variant) alignments were performed using the alignment program SCWRL [47]. The program MODELLER [39] was then used to create monomeric homology models of each IN based on the SCWRL sequence alignment and the WT I-TASSER structure. Model quality was assessed by Ramachandran analysis and based on root mean square deviation (RMSD) of the global homology structure from the PFV lead template using the RCSB PDB Protein Comparison Tool [48].

### Creation of a dimeric IN model

A dimeric model of CRF02_A/G IN was created by aligning a second monomer to the B chain of The PFV structure 3OY9. All non-aligned residues were deleted to yield only a partially resolved outer monomer as observed in PFV IN dimeric and tetrameric structures [14, 19]. Mn^2+^ and Zn^2+^ ions from PFV were retained in the dimeric structure of the CRF02_A/G IN to aid in docking if needed. PyMOL [49] was used for most protein visualizations.

### Compound library docking

Thirty randomly selected compounds from the ZINC database were screened as possible IN inhibitors [50] that primarily target IN:DNA binding and IN:IN dimerization interfaces. The preparation of receptor and ligand residues and docking simulations was performed using the PyRx [51] implementation of AUTODOCK Vina [52]. The top 5 hits based on calculated binding energies were further analyzed based on their binding interface, strength, and similarity to published INIs for potential future biochemical validation. Docking was also performed using the well characterized IN DNA binding inhibitor, FZ41 (CID 5481653), and a putative IN DNA binding inhibitor, HDS1 (CID 10814237-nigranoic acid). The compound HDS1 was investigated further.

### Antiviral activity of HDS1 measured by RT activity and quantitative PCR

The effect of HDS1 on reverse transcriptase activity present in culture supernatants was measured using a tritiated thymidine triphosphate based assay as previously described [53]. The effects of HDS1 on production of HIV-1 early and late reverse transcripts were measured by qPCR as previously described [54], with RAL and zidovudine (AZT) as controls.

### Biochemical evaluation of the impact of HDS1 on IN

The inhibitory impact of HDS1 on IN protein was assessed by three discrete reactions; strand transfer, 3' processing and LTR-DNA binding. The strand transfer assay was performed with fixed enzyme and substrate quantities in the presence of dose-ranging concentrations of HDS1. All assay conditions were as previously described [22]. The 3' processing assay was performed as previously described [55] in the presence of dose ranging concentrations of HDS1. The effect of dose-ranging concentrations of HDS1 on binding of LTR DNA to IN protein was assessed as previously described [56]. To test if HDS1 has intercalative DNA-binding activity, an ethidium bromide (EtBr) displacement assay was carried out as reported previously [57]. Briefly, a solution of EtBr at 1.26 μM was pre-incubated for 10 min at room temperature with a plasmid DNA or target DNA (1 μM) in a reaction buffer (2 mM HEPES, 10 μM EDTA, 9.4 mM NaCl, pH 7.0). After the incubation, test compound was added into the DNA–EtBr mixture at different concentrations ranging from 0.01–1000 μM. The fluorescence intensity of each mixture was measured (ex. at 544 nm, em. at 590 nm) by a FLUOStar Optima plate reader (BMG Labtech).

## Results and Discussion

In this study, we created several models of HIV IN using available free software and optimised and created a template model of HIV-1 CRF02 AG IN that could be used for drug screening and/or variant protein modeling.

Models 1–5 were created utilising HHpred (Models 1–3) (S1–S4 Figs) and PHYRE2 (Models 4 and 5) (S5 and S6 Figs). Some of these models are shown in the supplementary material. Models 1–3 did not have good 3-dimensional scores by either Verify3D and or ANOLEA (S1–S3 Figs). Models 4 and 5 were based primarily on HIV partial structures (Model 4) or PFV structures, respectively (Model 5). These models aligned primarily with the template HIV or PFV IN protein but not vice-versa (S4 Fig) and were therefore not further studied, highlighting the importance of selecting the right program for modeling of HIV proteins. The databases and methods used by these two programs differed slightly from I-TASSER which scanned the protein database (PDB) as well as allowed a certain level of user control. HHpred, for example, primarily scans pFam databases, but the classification of IN proteins across species, especially for PFV IN, is incomplete in most cases and, accordingly, PFV structures were mostly ignored in multiple sequence threading alignments.

The final IN MTM model (model 6) (Fig 1B) was created by multi-template threading utilizing the I-TASSER server [58] with the PFV lead template 3OY9. This allowed not only for the creation of a global model based primarily on the structure of PFV IN, but also allowed the folding of sequence fragments to be driven primarily by the multiple structural fragments of available HIV IN in the PDB, leading to a more representative structure. PFV IN structures are the only full-length IN structures that have bound DNA and are also the only structures that have bound drug. However, HIV structures should not be ignored because HIV IN has only ~20% sequence homology with PFV IN. Model 6, like the PFV template, has a mainly helical CCD domain with a largely disordered CTD domain and an elongated NTD domain. The domain orientation in the models was similar to that of the PFV crystal structure [14] and previously modelled HIV integrase models [19, 20].

Alignment of Model 6 with either of 1EX4 and 3OY9 yielded very good RMSD scores for the aligned regions and the CTD of the model followed a similar trajectory to that of PFV (Fig 1B). Verify3D plots indicate that the CCD-CTD portion of model 6 mostly have good 3-D structure with the NTD being poor to fair (Fig 1C). This is probably the major reason for an underwhelming score of 60.93, that is nonetheless higher than those of all the previous models studied with the exception of model 4 (89.8). The score of model 4 was even higher than its lead template, 1EX4 (73.02).

Comparing the Ramachandran plots of the two crystal structures 1EX4 and 3OY9 to model 6, it is evident that model 6 and 1EX4 have fewer residues in favored regions, with more in allowed and disallowed regions (84.8, 9.4, 6.3 and 80.8, 14.4, 4.8, respectively) than 3OY9 (96.7, 3.3, 0.0) (S7 Fig). Side-chains of residues in disallowed regions have steric clashes with other residues and are not likely in a steady state orientation. This points to more general disorder in HIV-1 IN relative to PFV and also implies that the lower structural confidence scores for model 6 are due to the HIV templates rather than the PFV templates. Model 6 was used as a template to create model 7 by single template threading utilizing the ProtMod server (http://ffas.burnham.org/protmod-cgi/protModHome.pl). This resulted in a structurally improved model that had 94.7% of peptide bonds in favoured regions, 4.1% in allowed regions and 1.2% in outlier regions (S7 Fig). Monomer B of CRF02_A/G IN was created as described in the methodology (Fig 1D) and an overlay of dimeric HIV-1 CRF02_A/G IN with the PFV structure 3OY9 is shown (Fig 1E).

### Docking simulations

**Fig 2:**
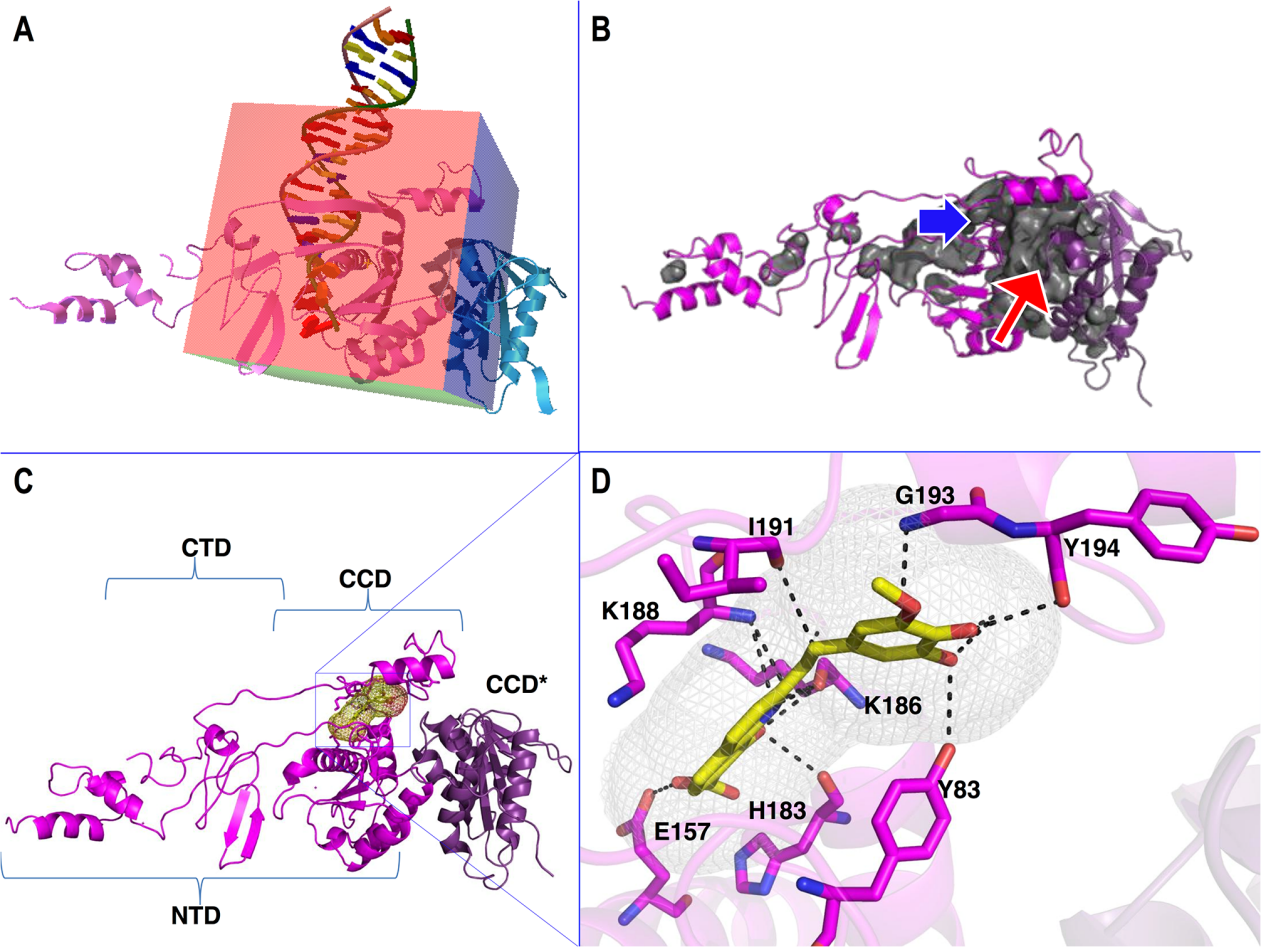
Location of docking grid box and docking of FZ41 to the IN dimer. (A) Grid box size and coordinates overlapped with the DNA binding domain as well as the dimerization interface. (B) Pockets within the dimeric model at the DNA interface (blue arrow) and at the dimer interface (red arrow). (C) FZ41 (yellow stick structure within grey mesh) binds within the DNA binding domain. Domains spanning regions corresponding to NTD, CCD, CTD for monomer A and CCD* for monomer B are indicated. (D) Interactions of FZ41 with IN residues. Docking simulations were performed utilizing AutoDock Vina [52] on a PyRx platform [51]. All image processing was done using PyMOL [59]. Solvent accessible pockets with a radius larger than 5Å are shown and colored grey. In the figure, the two monomers of the dimer are represented by different shades of magenta. CPK standard coloration is used for stick structural representations. Putative interacting atoms are indicated by a black dashed line.

Similar to most DNA binding proteins and especially those that have to undergo considerable modification upon substrate binding, IN has large solvent-accessible pockets; hence, there are potential binding pockets for inhibitory compounds. The program PyRx [51] was used for docking simulations with a 50Å x 50Å x 50Å grid box that encompassed the active site as well as the DNA binding and dimerization interfaces (Fig 2A). Thirty randomly chosen compounds from the ZINC database were utilised as ligands (S8 Fig). Compounds that docked near the blue arrow (Fig 2B) were considered to bind at the IN-DNA interface while compounds that docked near or around the red arrow were deemed to be dimerization modulators (Fig 2B). As has been previously published for PFV [19] and other HIV homology models, a CCD:CCD interaction defines the dimer interface (Fig 2C) while bound viral DNA contacts all three domains (Fig 2A).

**Fig 3:**
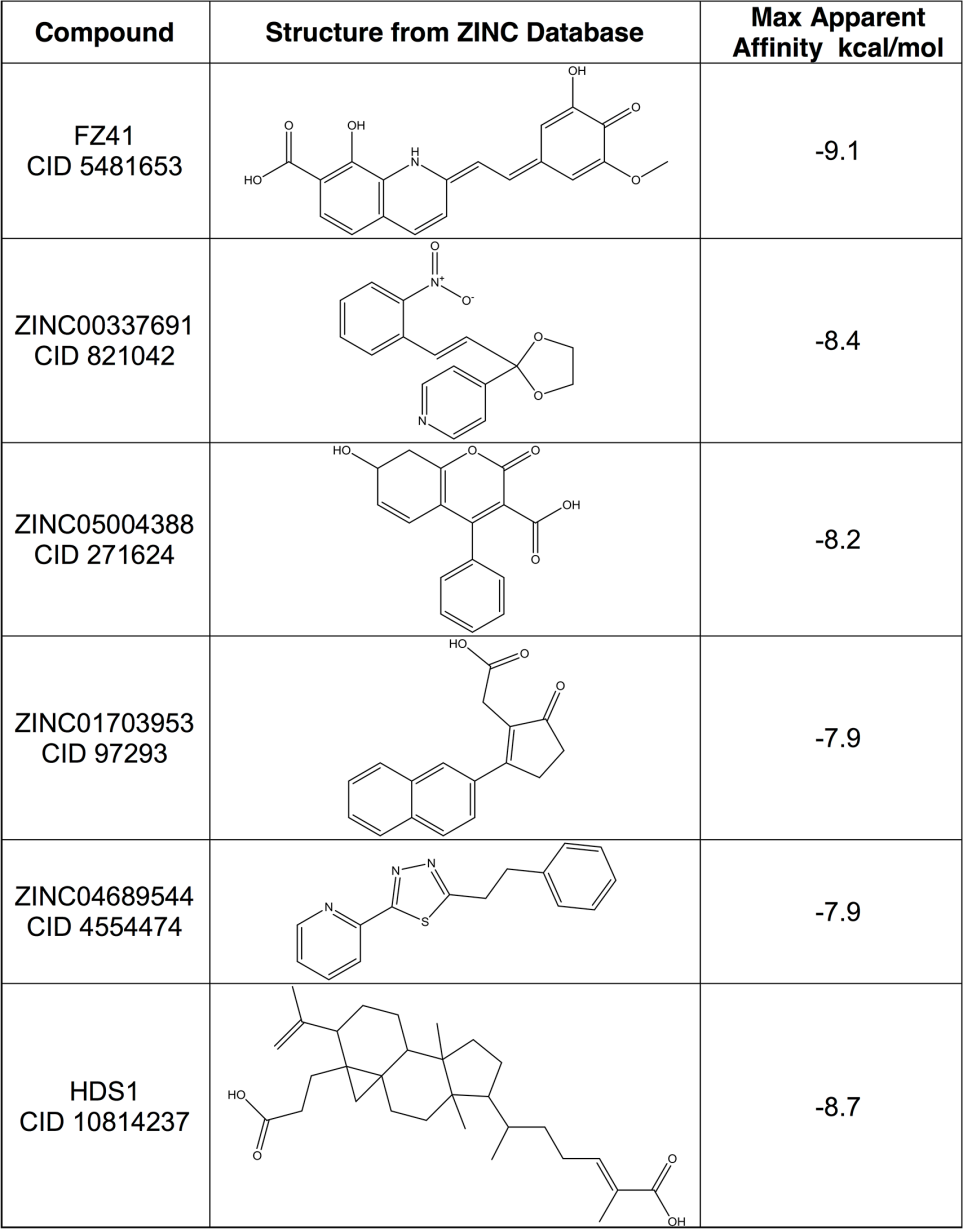
Best-docked compounds to the dimeric CRF02_A/G IN model.

The ZINC compounds screened and the apparent affinity calculations of the top 100 docked poses are shown in S8 Fig The highest binding energy calculated was -8.4 kcal/mol, calculated for ZINC00337691 (CID 821042). The apparent binding affinity for top poses of FZ41 and HDS-1 were -9.1 kcal/mol and -8.7 kcal/mol, respectively (Fig 3), while the apparent affinity for the 100th best docked ZINC compound orientation was -4.5 kcal/mol (S1 Fig). The chemical structures of the top five ZINC hits are presented (Fig 3).

Published reports of FZ41 implied that this inhibitor acted at a post-RT to early-integration stage of the viral cycle [60].We recently confirmed that this molecule inhibited viral replication and integration by decreasing IN binding to viral DNA [56]. In accordance with this result, FZ41 docked within the IN-DNA interface (Fig 2C and 2D) and formed hydrophilic interactions in this pocket with residues Y83, Y194, G193, I191, K188, E157 and H183. Of these residues, both K188 and H183 have been shown to be involved in viral DNA binding [40]; the charge at K188 has been shown as being critical for maintaining IN structural integrity and HIV infectivity in cell culture [61]. Residue G193 has also been shown to affect viral LTR specificity [62]. The location of FZ41 may also mean that it can have a modulating effect on IN quaternary structure, in addition to inhibition of DNA binding, since it also has an inhibitory effect on nuclear import [63]. The putative FZ41 binding domain overlaps with that previously described for a group of putative allosteric inhibitors of HIV IN [64].

**Fig 4:**
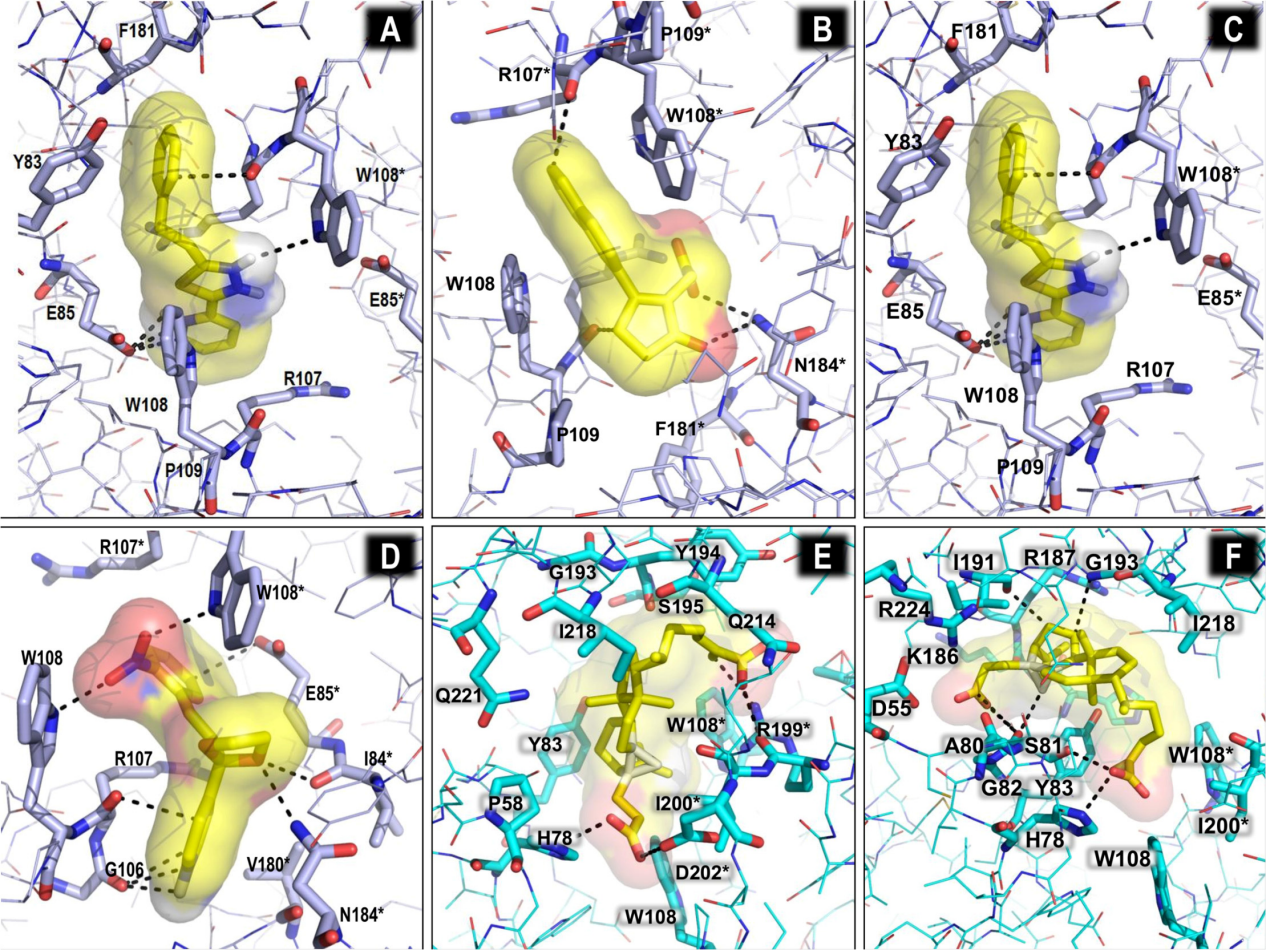
Screening of compounds for interaction with dimeric CRF02_AG IN in the absence of DNA ligands. Interactions of ZINC00337691 (A), ZINC01703953 (B), and ZINC04689544 (C) with residues at the dimer interface. (D) The binding interface of HDS1 spans the dimer (red oval) and DNA binding (blue oval) interfaces. (E) Binding of HDS1 at the dimer interface and (F) DNA interface. In all panels above, the docked compound is represented by yellow main-chain sticks, while the global structure is represented by lines or cartoons and interacting residues are shown as cyan stick structures. Standard CPK coloration is used for stick and line structures. Putative interacting atoms are indicated by a black dashed line.

The compound ZINC00337691 (CID 821042) docks into the IN:IN dimer-interface (Fig 4A). ZINC00337691 (4-[2-[(E)-2-(2-nitrophenyl)ethenyl]-1,3-dioxolan-2-yl]pyridine) may act to stabilize the dimeric complex, since it has interactions with both monomers. It also has hydrophilic interactions with the main chain carbonyls of G106, R107 and I84 as well as with the side chains of N184 of chain A. Residue W108 of both subunits forms both hydrophobic stacking interactions and electrostatic interactions with the nitrophenyl portion of the compound. The binding pocket is framed by the hydrophobic contributions from the aliphatic portions of the R107, E85, and V180 residues and the hydrophobic stacking interactions with the two W108 residues. The stabilization of IN dimeric structure has previously been reported for a small group of IN allosteric inhibitors called LEDGINs [5, 62]; even though ZINC00337691 does not appear to bind at the same location, it might stabilize the dimer as well as prevent movement of the protein structure and might therefore be active as a cross-sectional inhibitor in a similar manner as IN allosteric inhibitors [62]. Residues E85 and N184 seem to also coincide at least partially with the Rev binding interface (48) and this compound may also affect Rev regulation of IN nuclear import [65].

Docking of ZINC01703953 (2-(2-naphthalen-2-yl-5-oxocyclopenten-1-yl)acetic acid) (CID 97293) was into the same general location as that of ZINC00337691 at the dimer-interface (Fig 4B) but with a somewhat snugger fit. Binding of this compound appeared to be driven mostly by van der Waals interactions and shape complementarity because there were limited hydrogen bonding interactions. ZINC01703953 interacted with R107, W108, P109, F181 and N184 of monomer A and R107, W108 and P109 of monomer B. These residues are all implicated in Rev and distal DNA binding effects. ZINC01703953 also has a diketocarboxylic acid moiety and may possibly exhibit some strand transfer activity under appropriate circumstances. Another compound that docks into the inter-monomer interface, ZINC04689544 (2-(2-phenylethyl)-5-pyridin-2-yl-1,3,4-thiadiazole) (CID 4554474), is more elongated (Fig 4C), forming extensive van der Waals contacts with Y83, E85, R107, W108, P109 and F181 of monomer A and E85 and W108 of monomer B. There are also some hydrogen-bonding interactions with W108 and a salt-bridge with E85 of IN subunit A. The salt-bridge is likely a key driving force for these binding interactions. Additionally, the elongated hydrophobic nature of this molecule may cause it to occupy more space at the interface and it might be a potent modulator of IN activity.

The docking footprint of HDS1 (CID 10814237) on the dimer spanned both the DNA-interaction interface as well as the IN:IN dimer interface (Fig 4D). Three of the best five orientations docked closer to the dimer interface (Fig 4E) while two docked in a similar location to FZ41. These HDS1 docking interactions appeared to be driven by van der Waals interactions and shape complementarity with best docked affinity calculations of -8.7 kcal/mol and -8.2 kcal/mol, respectively.

### Characterization of the inhibitory impact of HDS1

**Fig 5:**
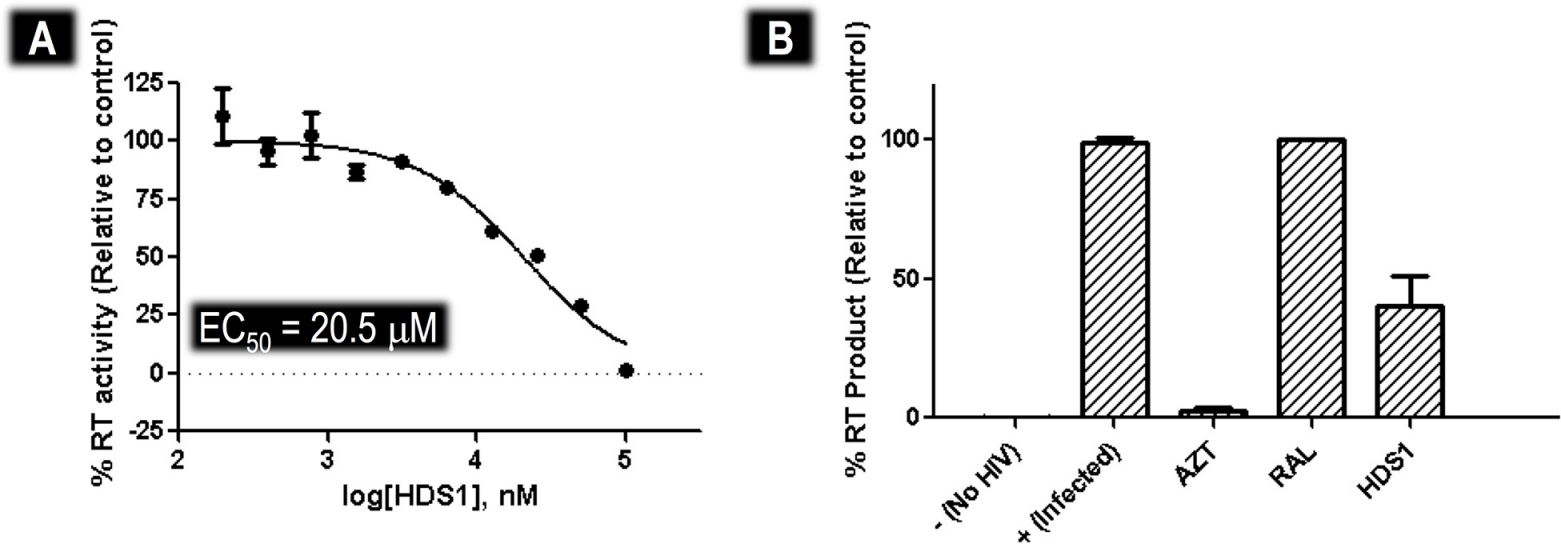
Inhibition of HIV-1 in MT-4 cell cultures by HDS1. (A) Dose-dependent inhibition of HIV-1 replication in cell cultures by HDS1. HIV-1 reverse transcriptase activity in cell culture fluids was measured using a tritiated thymidine triphosphate based assay as previously described [53]. (B) Effect of AZT (1 μM), RAL(0.5 μM) and HDS1 (20 μM) on production of late HIV-1 reverse transcripts as measured by qPCR.

Although HDS1 inhibits IN DNA binding activity, its effect on viral replication, reverse transcription and/or integration has not been evaluated. Here we show in MT2 cell culture inhibition assays that HDS1 inhibited viral replication as measured by RT activity in cell culture with an half-effective concentration (EC_50_) of 20.5 μM (Fig 5A). When quantitative PCR (qPCR) was used to measure the effect of HIV inhibitors, zidovudine (AZT) fully suppressed production of late reverse transcripts, due to its role as a reverse transcription inhibitor, while RAL permitted a build-up of late RT transcripts, due to its role as a post-RT inhibitor. HDS1 also permitted a build-up of late RT transcripts but only to a level of 50% of that associated with RAL. This is also consistent with the reported activity of HDS1 as a weak RT inhibitor [66]. (Fig 5B).

**Fig 6:**
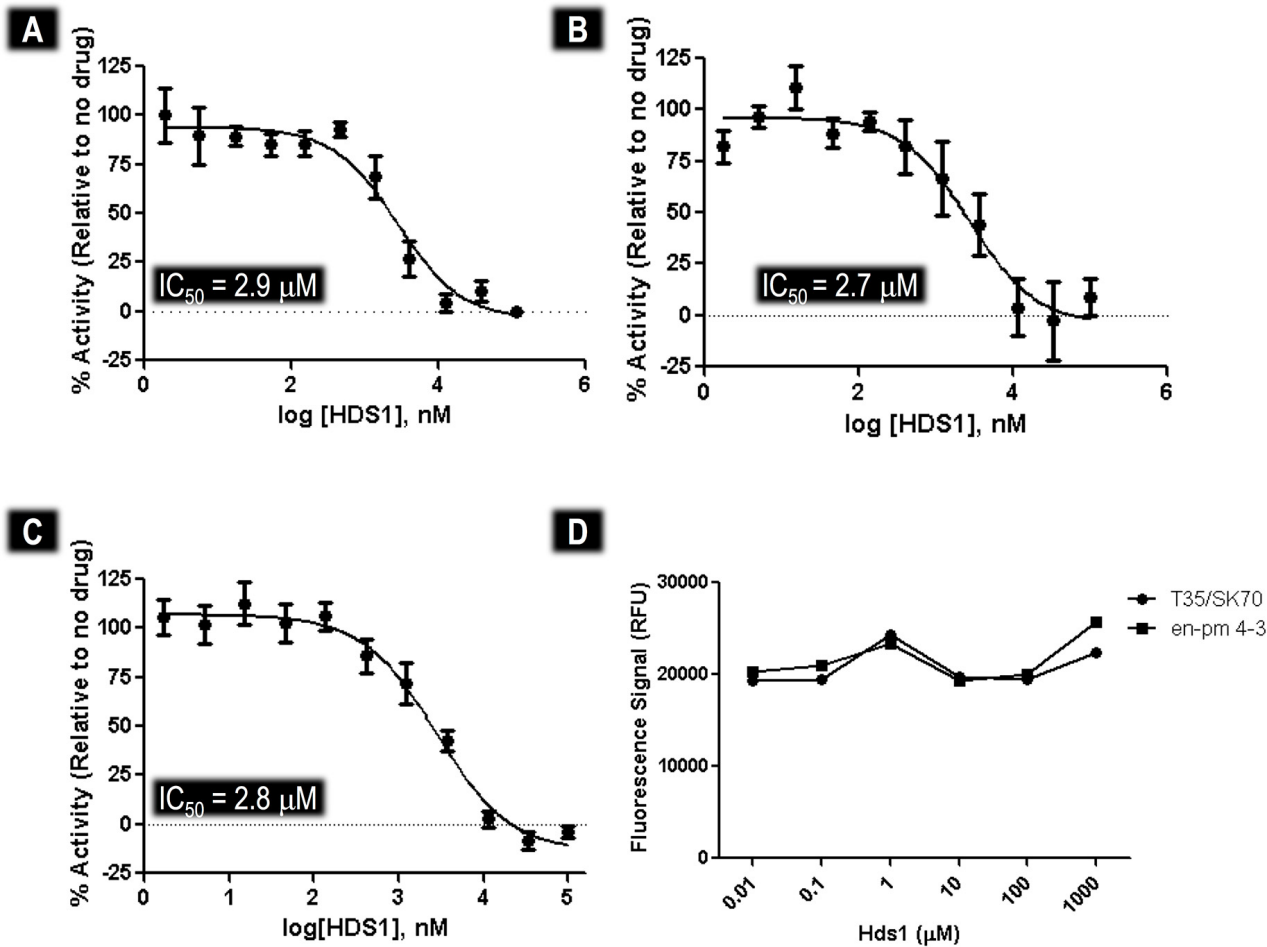
Biochemical Characterisation of HDS1 on purified HIV-1 IN enzyme. (A) Inhibition of the strand transfer reaction. (B) Inhibition of the 3' processing reaction. (C) Inhibition of the DNA binding activity of HIV integrase. (D) Test for the ability of HDS1 to bind to DNA.

Biochemical analysis of the effect of HDS1 on integration confirmed that it impacted DNA binding. The individual IC_50_s for inhibition of strand transfer (Fig 6A), 3' processing (Fig 6B) and integrase-DNA binding (Fig 6C) of HDS1 were 2.9 μM, 2.7 μM and 2.9 μM, respectively. Given that both 3' processing and strand transfer require DNA binding to take place and given that the IC_50_s for inhibition of these reactions were neither additive nor synergistic with inhibition of DNA binding, we conclude that HDS1 blocks integration primarily by inhibition of DNA binding.

However, our docking studies on HDS1 (Fig 4C–4E) did not show a direct interaction of HDS1 with DNA in the DNA binding trough, as one would expect in the case of a DNA binding antagonist. We therefore investigated whether HDS1 could affect the ability of ethidium bromide (EtBr) to bind to double- stranded DNA (Fig 6D). An EtBr displacement assays showed that the addition of HDS1 did not result in decrease in fluorescence intensity, suggesting that it was unable to displace EtBr. These results demonstrate that HDS1 did not directly interact with DNA.

## Conclusions

We have presented a comprehensive method for creation of viable HIV IN models based on the partial HIV crystal structures as well as full-length PFV IN structures. These models were in good agreement with the PFV crystal structures as well as two published HIV integrase models [19, 20]. They also did not deviate from DNA:IN architecture as proposed by Kessl and colleagues [21], despite the fact that this group studied DNA-bound tetramers in the presence and absence of the integrase ligand LEDGF/p75 and showed alternate quaternary assembled structures. We have previously utilized these models to investigate the binding of INSTIs to IN and the impact of resistance mutations on enzyme function [22, 67]. Here, we investigated the ability of the models to screen for compounds that bind at the viral LTR interaction domain or at the IN:IN dimerization domain. Given that multiple partial structures of HIV IN have variable structural conformations[4] and different observed dimerization phases, we preferred the quaternary arrangement that is most probable in the active PIC, based on the available structures of PFV IN [14–17, 19]. By utilizing freely available software and screening the ZINC database, we demonstrated the utility of IN models to screen for novel inhibitors using compound databases.

The compound ZINC05004388 (7-hydroxy-2-oxo-4-phenyl-7,8-dihydrochromene-3-carboxylic acid) (CID 271624) (Fig 3) had structural similarity to a class of IN inhibitors that show clinical potential, i.e. non catalytic site IN inhibitors (NCINIs) or LEDGINs, named for their ability to block interaction of IN with its cellular tethering factor LEDGF [68]. Although our models might not have selected LEDGIN-type molecules [69], ZINC05004388 (CID 271624) binds at the same general location as does ZINC01703953 (not shown). Given that LEDGINs have been reported to inhibit IN at multiple steps of the integration process and viral life cycle, this may be an indication of the ability of these compounds to bind at more than one site within IN or to act at different steps of integration and the viral life cycle [70, 71]. Similar to most selected allosteric inhibitors of IN, the ZINC compounds that had the highest affinity calculations were hydrophobic and possessed significant ring structures joined by flexible linkers with isolated hydrophilic/charged moieties.

**Fig 7:**
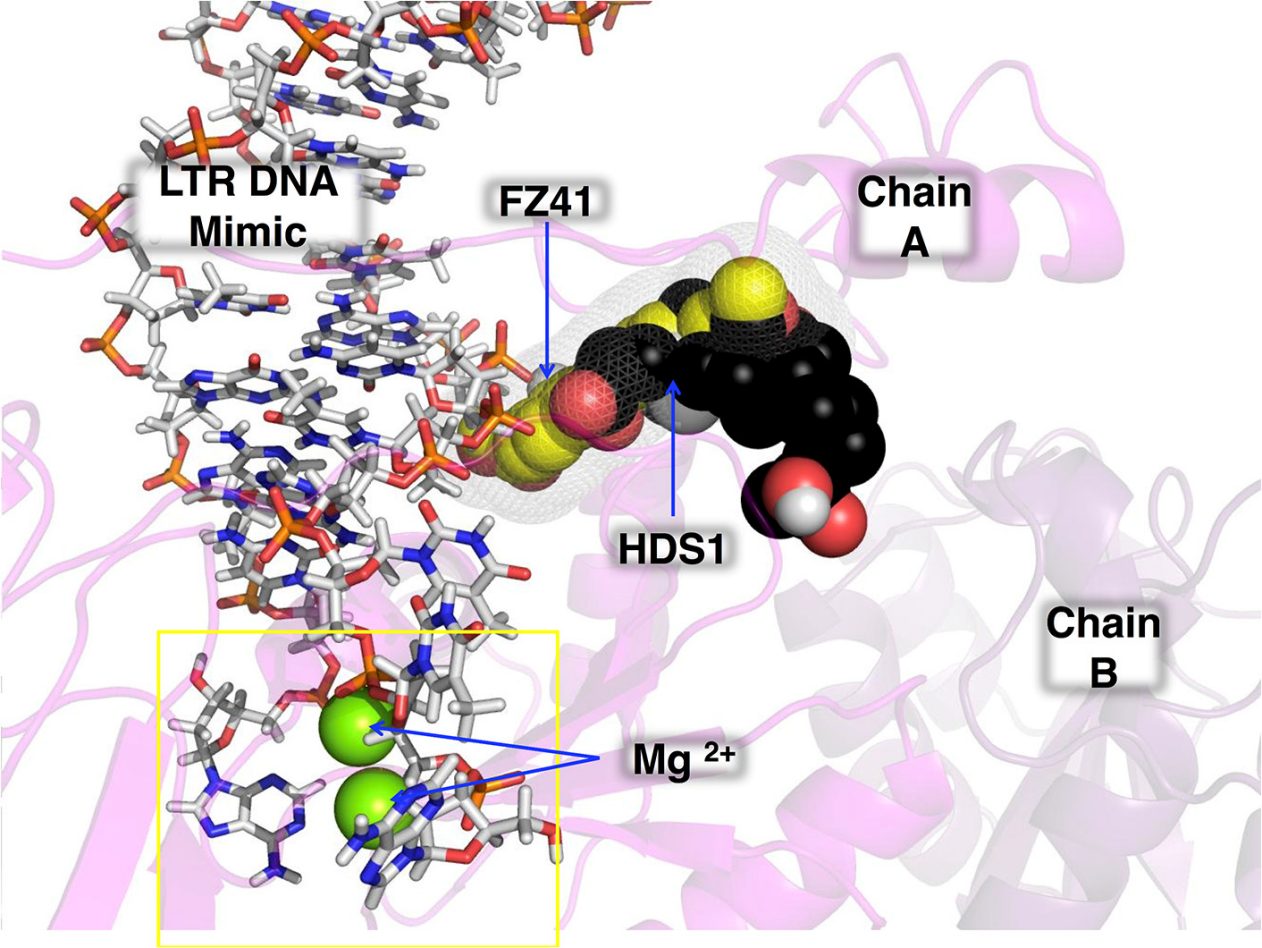
FZ41 and HDS1 may directly inhibit DNA binding to integrase. The HDS1 docked structure (shown as a spherical structure with black carbon atoms) (Fig 4F) was overlaid onto the FZ41 structure (shown as a spherical structure with yellow carbon atoms) in Fig 2D together with DNA (stick structure with white carbon atoms) coordinates from the PFV 3OY9 structure. The active site is indicated by a yellow rectangle. The two monomers of the dimer are represented by different shades of magenta. All other coloration is based on the CPK standard [18].

Our dimeric IN models confirmed that a compound that we previously selected using a DNA-binding screen, i.e. HDS1, binds at a similar location to a well characterized DNA binding inhibitor, FZ41 (Fig 7). This region of the IN dimer is important for DNA binding and activity (Fig 4D) but is not the target of any approved drug. Virological and biochemical characterization of HDS1 further confirmed that it exhibits a similar activity profile as FZ41 [60]. The binding of either of these compounds to this site most likely inhibits DNA binding through direct steric inhibition and/or altered inter-residue interactions. The elucidation of this unexploited pocket in HIV IN may potentially yield new antiviral compounds in the future.

## Supporting Information

S1 Fig Ten of the major 15 structural homologues of CRF02_A/G integrase identified by HHpred and aligned using T-COFFEE [72]. In alignment, key structural and functional features are indicated in highlighted regions; Zn^2+^ binding domains are indicated by green rectangles and labeled H2 and C2 for relevant portions of the H2C2 motif. CCD catalytic residues are boxed in blue and labeled D,D and E as appropriate. Key residues involved in INSTI drug resistance are highlighted in red boxes reflecting positions 92, 118, 140, 143, 148, 155 and 263, respectively, based on previously published data [73, 74].
(PPTX)

S2 Fig Automated modeling of CRF02_A/G IN. (Figure A) The globular model 1 that was generated and (Figure B) the alignment of the model with the 'best' two possible templates, HIV (dark green) and PFV (cyan) using HHpred. RMSD values are indicated for the structurally aligned portions only. The quality of the model was evaluated using (Figure C) VERIFY 3D and (Figure D) ANOLEA algorithms.
(PPTX)

S3 Fig Modeling of CRF02_A/G IN based on the major 10 structural and sequence homologues. (Figure A) Alignment of model 2 (green) with the 'best' two possible templates, HIV (dark green) and PFV (cyan). RMSD values are indicated for the structurally aligned portions only. The quality of model 2 was verified by (Figure B) Verify3D and (Figure C) ANOLEA.
(PPTX)

S4 Fig HHpred alignment used to create model 3 with key features highlighted. The H2C2 motif is indicated by blue highlighted boxes, catalytic residues with black arrows, and locations important for INSTI resistance with red circles. Acidic residues are colored red, basic residues are colored blue, hydrophobic residues are colored green and hydrophilic residues black. The predicted secondary structure of the query (Q ss_pred) is also shown with the predicted secondary structure of the template (T ss_pred) and the actual secondary structure of the template (T ss_dssp; "H" denotes helices, "C" coils, "E" extended β-strand). Sequence conservation between the two sequences is shown in two manners; any consensus residues between the template and query sequences are linked by a"|", conservative substitutions are linked with a "+" and non-conservative substitutions with ".". In the consensus sequence (Q Consensus), "~" denotes non-consensus residues. Gaps in the alignment are represented by "-". Uppercase letters are strong trends and lowercase letters represent lower confidence trends.
(PPTX)

S5 Fig Modeling of the CRF02_A/G integrase model 4 (Figure A) and (Figure B) the alignment of the model with the 'best' two possible templates. RMSD values are indicated for the structurally aligned portions only.
(PPTX)

S6 Fig Structural alignment of model 5 (yellow) with 3OY9 (cyan) and 1EX4 (dark green). (PPTX)

S7 Fig Ramachandran plot analysis of models 6 and 7 compared to the two lead templates. (PPTX)

S8 Fig Summary comparison of the top 100 binding energies and their relative displacements as calculated using Autodock Vina. (PPTX)
