# Supplementary material for: Structural Studies of the HIV-1 Integrase Protein: Compound Screening and Characterization of a DNA-Binding Inhibitor
Source: PLoS One. 2015 Jun 5;10(6):e0128310. doi: 10.1371/journal.pone.0128310 (PMC4457863; doi:10.1371/journal.pone.0128310)
Supplement: S1 Fig — In alignment, key structural and functional features are indicated in highlighted regions; Zn2+ binding domains are indicated by green rectangles and labeled H2 and C2 for relevant portions of the H2C2 motif. CCD catalytic residues are boxed in blue and labeled D,D and E as appropriate. Key residues involved in INSTI drug resistance are highlighted in red boxes reflecting positions 92, 118, 140, 143, 148, 155 and 263, respectively, based on previously published data [73, 74]. (PPTX) [file pone.0128310.s001.pptx]

## Slide 1
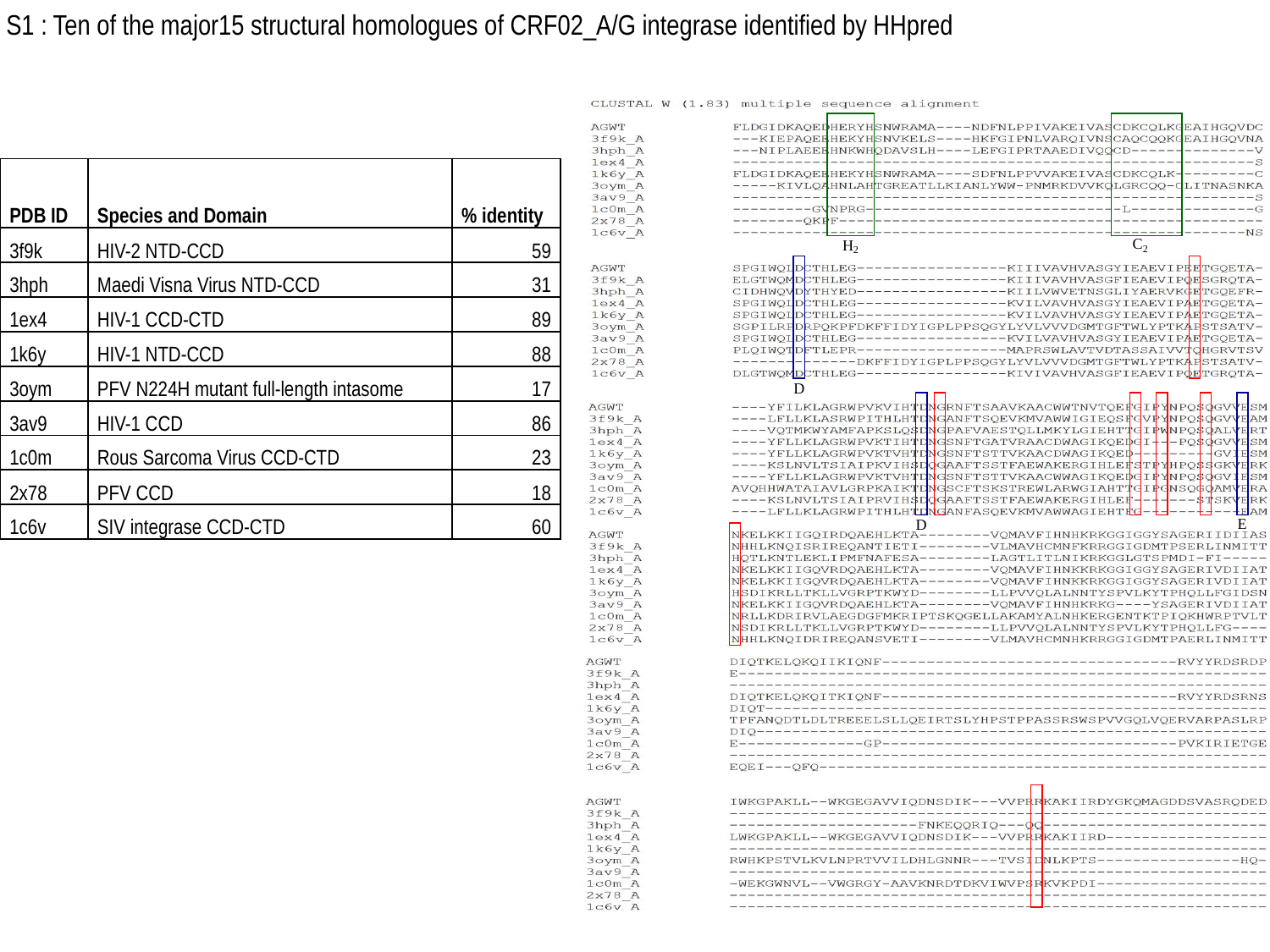

S1 : Ten of the major15 structural homologues of CRF02_A/G integrase identified by HHpred
| PDB ID | Species and Domain | % identity |
| --- | --- | --- |
| 3f9k | HIV-2 NTD-CCD | 59 |
| 3hph | Maedi Visna Virus NTD-CCD | 31 |
| 1ex4 | HIV-1 CCD-CTD | 89 |
| 1k6y | HIV-1 NTD-CCD | 88 |
| 3oym | PFV N224H mutant full-length intasome | 17 |
| 3av9 | HIV-1 CCD | 86 |
| 1c0m | Rous Sarcoma Virus CCD-CTD | 23 |
| 2x78 | PFV CCD | 18 |
| 1c6v | SIV integrase CCD-CTD | 60 |
