# Supplementary figures and images for: Structural Studies of the HIV-1 Integrase Protein: Compound Screening and Characterization of a DNA-Binding Inhibitor
Source: PLoS One. 2015 Jun 5;10(6):e0128310. doi: 10.1371/journal.pone.0128310 (PMC4457863; doi:10.1371/journal.pone.0128310)

## Slide 1
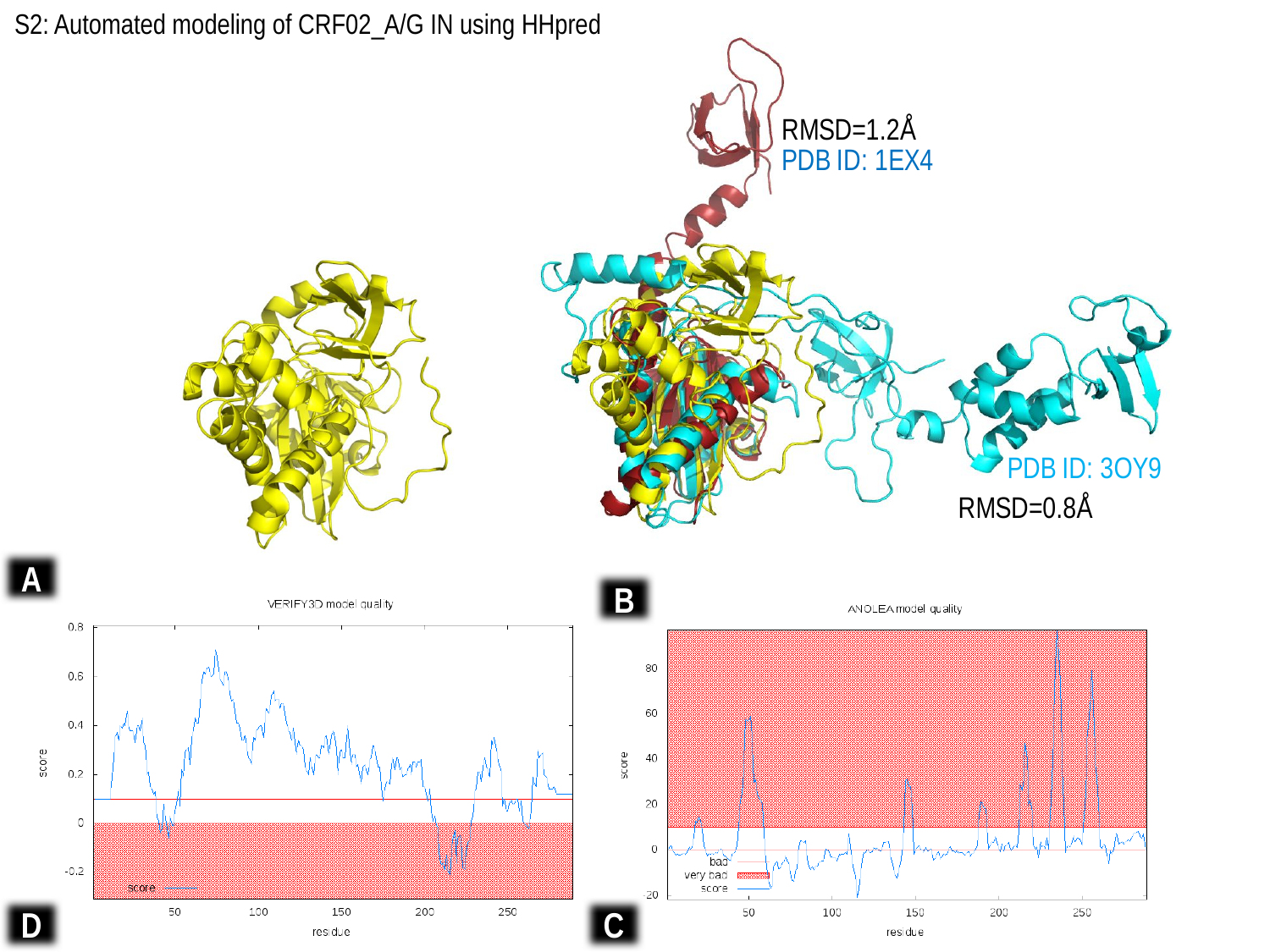

S2: Automated modeling of CRF02_A/G IN using HHpred
A
B
D
C

Supplement: S2 Fig — (Figure A) The globular model 1 that was generated and (Figure B) the alignment of the model with the 'best' two possible templates, HIV (dark green) and PFV (cyan) using HHpred. RMSD values are indicated for the structurally aligned portions only. The quality of the model was evaluated using (Figure C) VERIFY 3D and (Figure D) ANOLEA algorithms. (PPTX) [file pone.0128310.s002.pptx]

## Slide 1
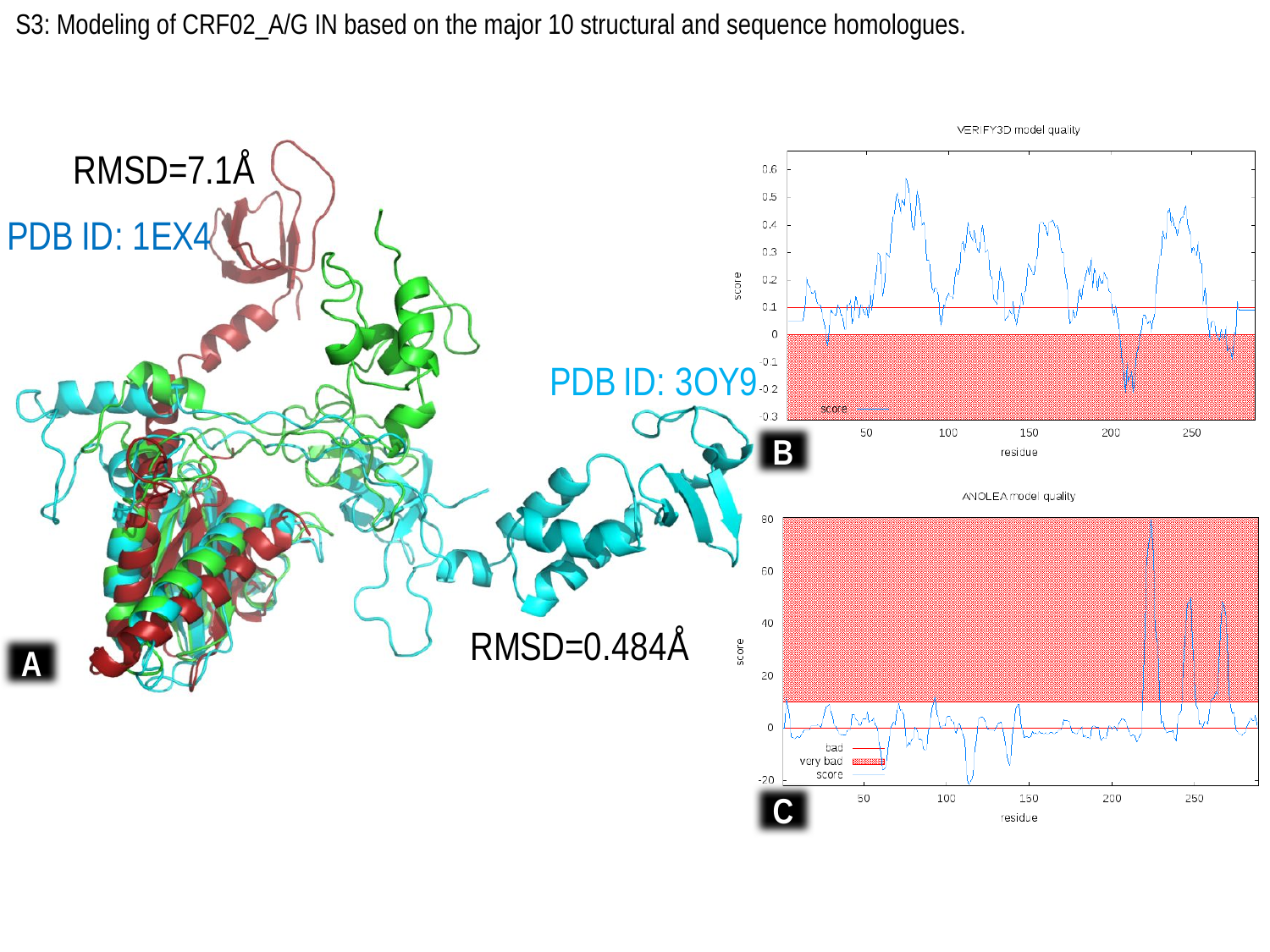

S3: Modeling of CRF02_A/G IN based on the major 10 structural and sequence homologues.
B
A
C

Supplement: S3 Fig — (Figure A) Alignment of model 2 (green) with the 'best' two possible templates, HIV (dark green) and PFV (cyan). RMSD values are indicated for the structurally aligned portions only. The quality of model 2 was verified by (Figure B) Verify3D and (Figure C) ANOLEA. (PPTX) [file pone.0128310.s003.pptx]

## Slide 1
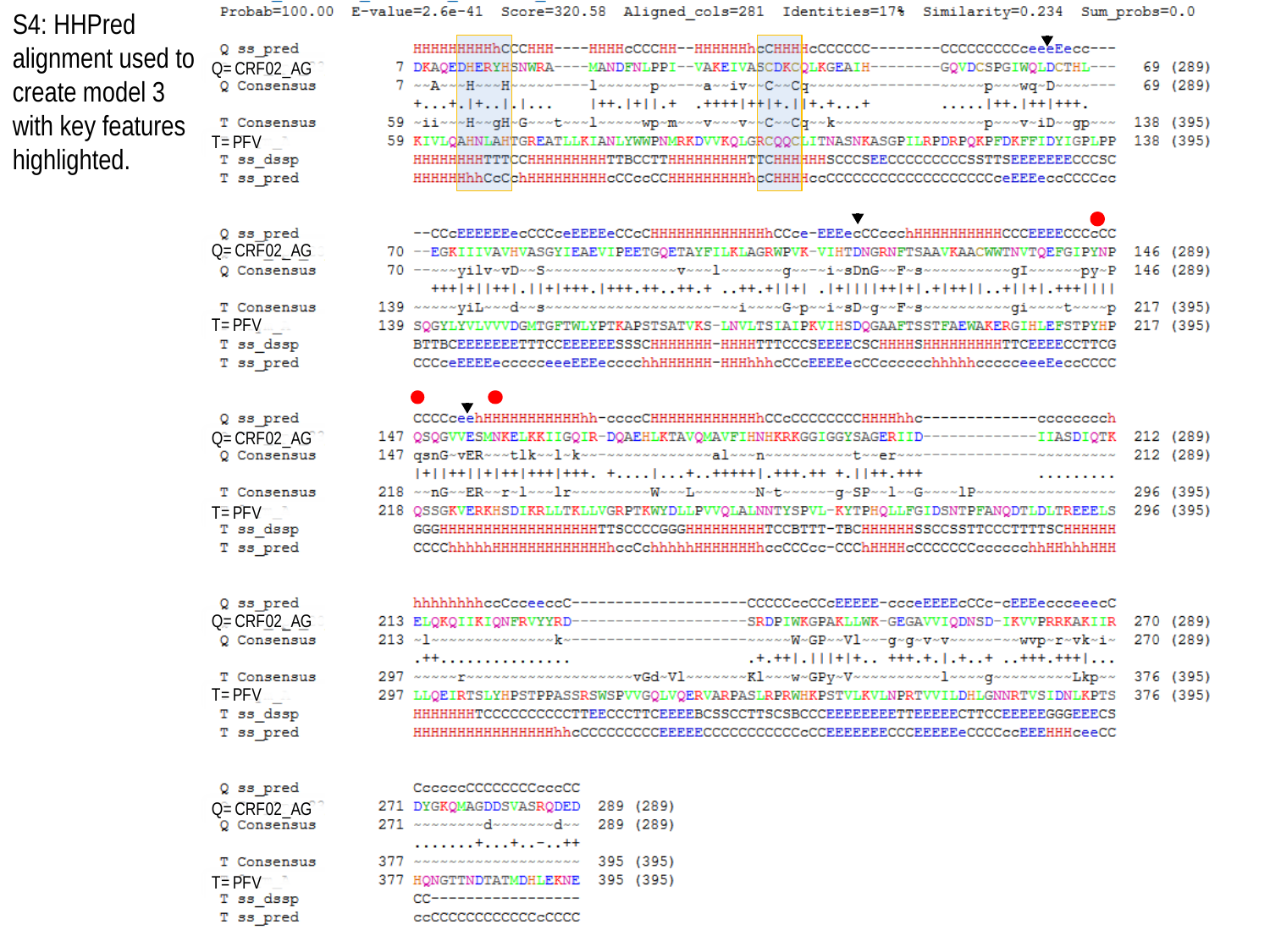

S4: HHPred alignment used to create model 3 with key features highlighted.

Supplement: S4 Fig — The H2C2 motif is indicated by blue highlighted boxes, catalytic residues with black arrows, and locations important for INSTI resistance with red circles. Acidic residues are colored red, basic residues are colored blue, hydrophobic residues are colored green and hydrophilic residues black. The predicted secondary structure of the query (Q ss_pred) is also shown with the predicted secondary structure of the template (T ss_pred) and the actual secondary structure of the template (T ss_dssp; "H" denotes helices, "C" coils, "E" extended β-strand). Sequence conservation between the two sequences is shown in two manners; any consensus residues between the template and query sequences are linked by a"|", conservative substitutions are linked with a "+" and non-conservative substitutions with ".". In the consensus sequence (Q Consensus), "~" denotes non-consensus residues. Gaps in the alignment are represented by "-". Uppercase letters are strong trends and lowercase letters represent lower confidence trends. (PPTX) [file pone.0128310.s004.pptx]

## Slide 1
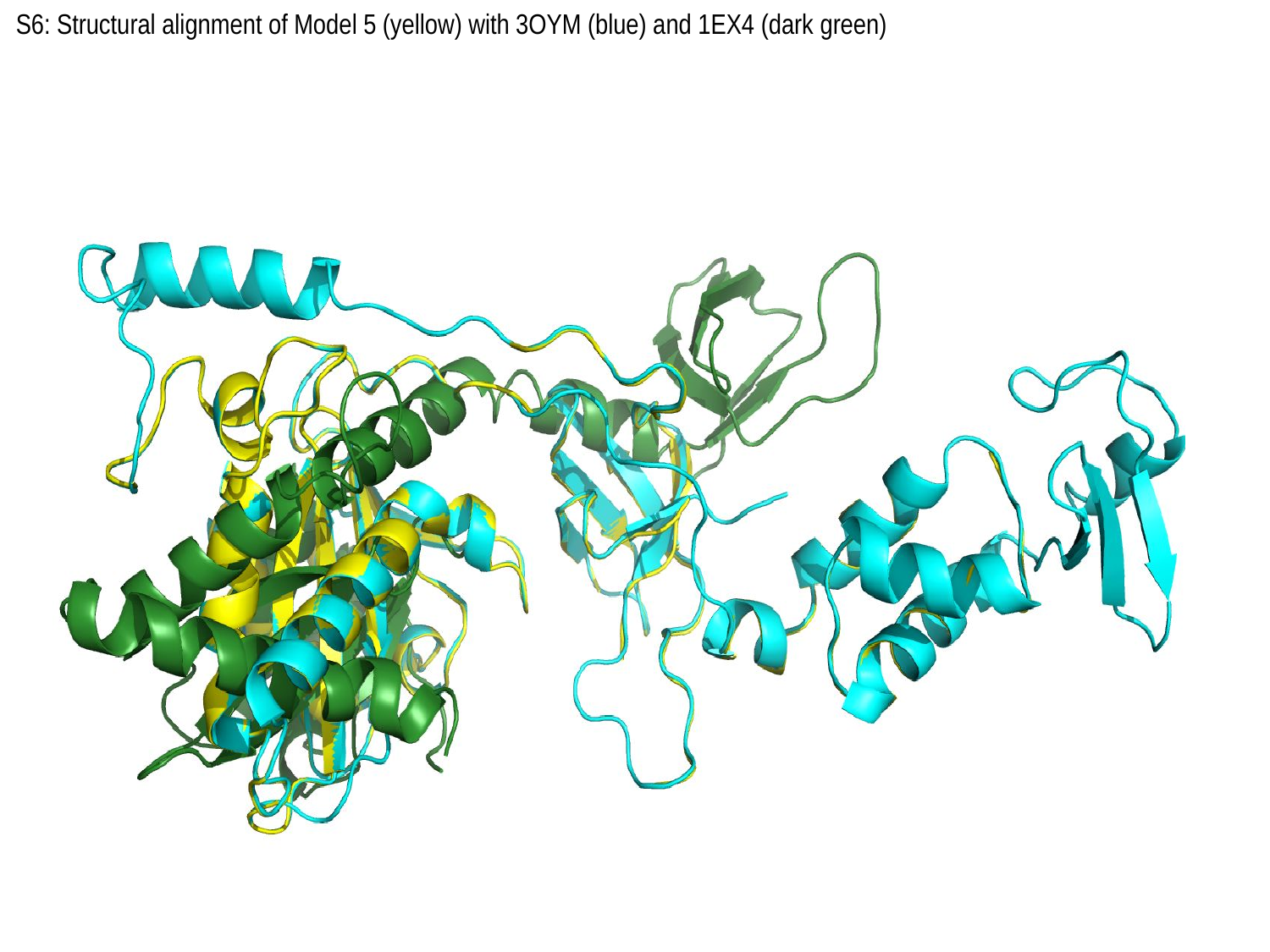

S6: Structural alignment of Model 5 (yellow) with 3OYM (blue) and 1EX4 (dark green)

Supplement: S6 Fig — (PPTX) [file pone.0128310.s006.pptx]
