# Supplementary material for: Structural Studies of the HIV-1 Integrase Protein: Compound Screening and Characterization of a DNA-Binding Inhibitor
Source: PLoS One. 2015 Jun 5;10(6):e0128310. doi: 10.1371/journal.pone.0128310 (PMC4457863; doi:10.1371/journal.pone.0128310)
Supplement: S5 Fig — RMSD values are indicated for the structurally aligned portions only. (PPTX) [file pone.0128310.s005.pptx]

## Slide 1
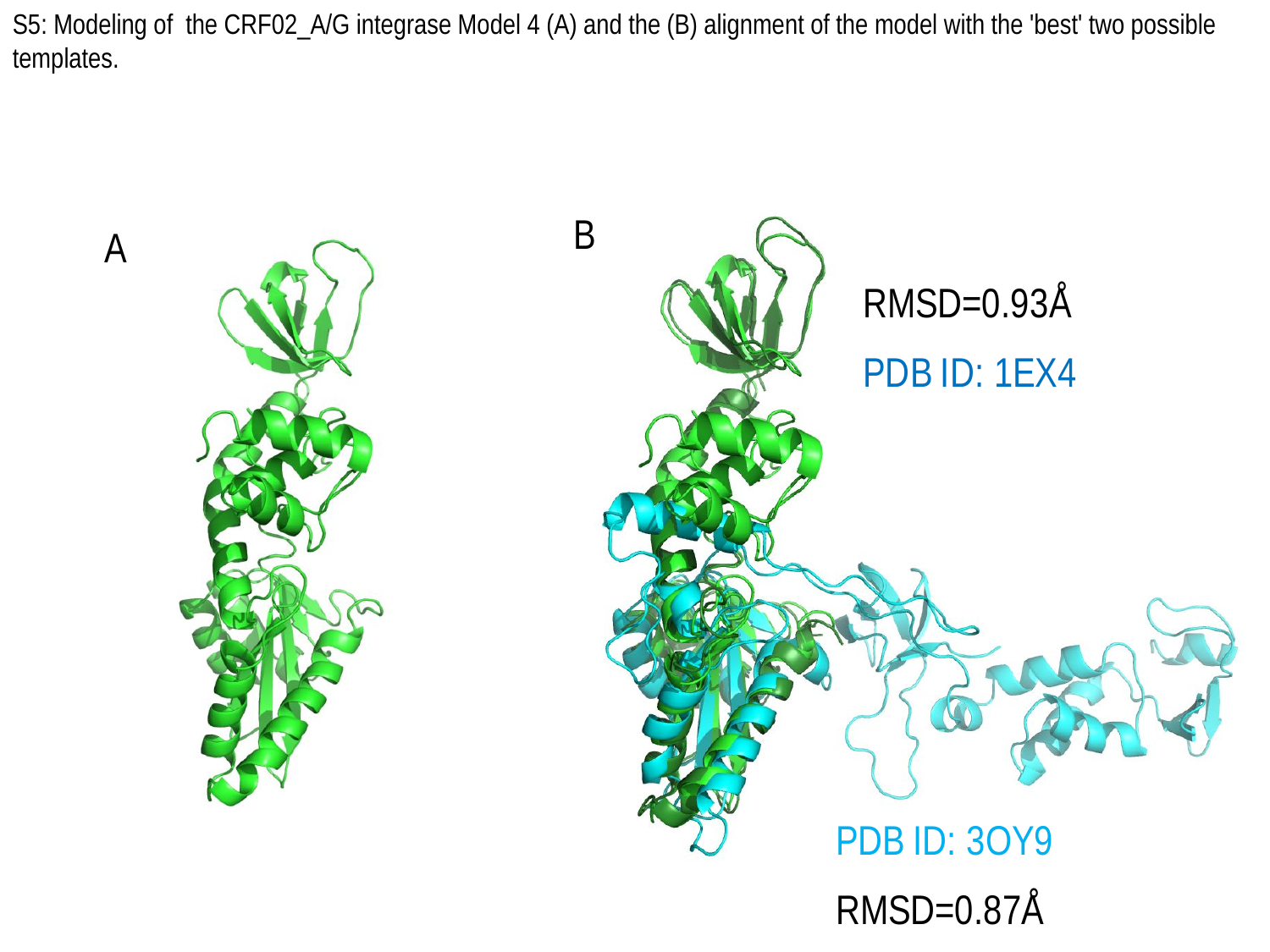

S5: Modeling of the CRF02_A/G integrase Model 4 (A) and the (B) alignment of the model with the 'best' two possible templates.
