# Supplementary material for: Structural Studies of the HIV-1 Integrase Protein: Compound Screening and Characterization of a DNA-Binding Inhibitor
Source: PLoS One. 2015 Jun 5;10(6):e0128310. doi: 10.1371/journal.pone.0128310 (PMC4457863; doi:10.1371/journal.pone.0128310)
Supplement: S7 Fig — (PPTX) [file pone.0128310.s007.pptx]

## Slide 1
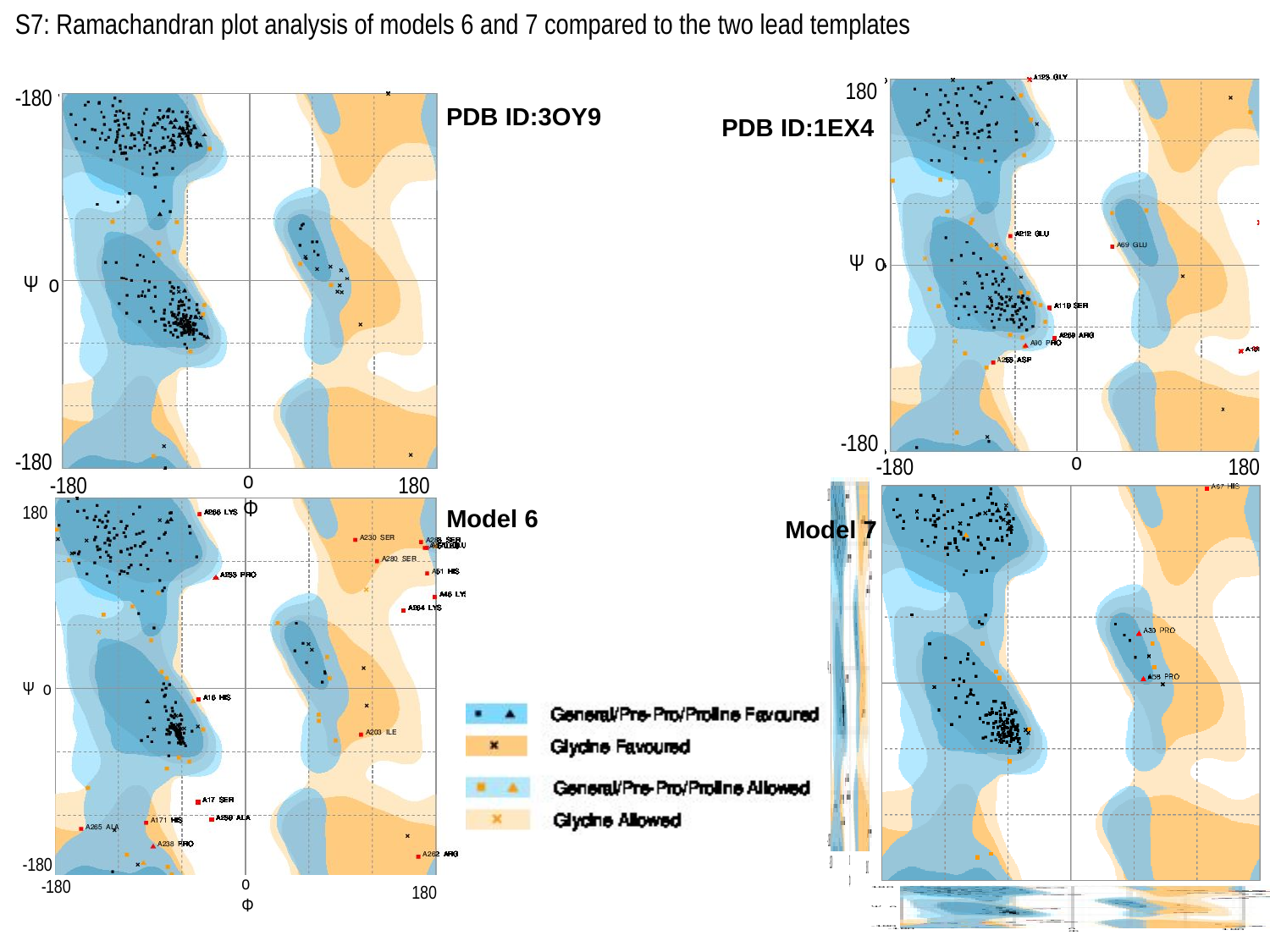

S7: Ramachandran plot analysis of models 6 and 7 compared to the two lead templates
PDB ID:3OY9
PDB ID:1EX4
Model 6
Model 7
